# Control of Schwann Cell Myelination by DDR1 Receptor Tyrosine Kinase

**DOI:** 10.3390/ijms27115135

**Published:** 2026-06-05

**Authors:** Mengyuan Fan, Yuchen Sun, Ruyi Mei, Wenwen Lu, Xiaofeng Zhao, Aifen Yang, Mengsheng Qiu

**Affiliations:** 1 College of Life and Environmental Sciences, Hangzhou Normal University, Hangzhou 311121, China; 2024111010019@stu.hznu.edu.cn (M.F.); 2024111010031@stu.hznu.edu.cn (Y.S.); luwenwen@hznu.edu.cn (W.L.); xfzhao@hznu.edu.cn (X.Z.); 2 Perioperative and Systems Medicine Laboratory, Zhejiang University School of Medicine, National Clinical Research Center for Children and Adolescents’ Health and Diseases, Hangzhou 310052, China; ruyi_mei@zju.edu.cn

**Keywords:** DDR1, Schwann cells, differentiation, myelination, ERK

## Abstract

In peripheral nervous system (PNS), immature Schwann cells differentiate into two functionally distinct types of mature Schwann cells: myelinating Schwann cells, which establish a one-to-one relationship with large-diameter axons and initiate the complex myelination program that enables the rapid saltatory conduction of nerve impulses, and non-myelinating Schwann cells, which envelop multiple small-diameter axons without forming myelin, but support axon survival and maintain microenvironment homeostasis. Here, we demonstrate that discoidin domain receptor 1 (DDR1) signaling plays a pivotal role in Schwann cell maturation and peripheral nerve myelination. *Ddr1*^−/−^ mice of both sexes exhibited fewer myelinating Schwann cells and profound hypomyelination, reduced nerve conduction velocity, and apparent motor dysfunction. Expression of myelin markers was markedly reduced in the mutants accompanied by the formation of abnormal myelin ultrastructure resembling “onion bulbs”. Mechanistically, *Ddr1* deficiency impaired Schwann cell myelination through the marked hyperactivation of the MAPK/ERK signaling pathway. These findings establish DDR1 as a novel regulator of Schwann cell myelination and highlight its potential as a therapeutic target for demyelinating neuropathies.

## 1. Introduction

The peripheral nervous system (PNS) is a highly heterogeneous and essential component of the nervous system, serving as the primary conduit between the central nervous system (CNS) and the periphery. Its proper function is indispensable for sensory perception, motor control, and autonomic regulation [1]. The peripheral nerves are primarily composed of axons and Schwann cells (SCs), the supporting glial cells that generate the insulating myelin sheath, facilitating the rapid saltatory conduction of nerve impulses [2]. Schwann cell precursors originate from neural crest progenitor cells. During early postnatal development, they undergo a well-regulated radial sorting process, which segregates and individually ensheathes large-caliber axons (diameter > 1 μm in rodents) and bundles smaller axons into non-myelinating Remak fibers [3]. Beyond myelination, SCs ensure neuronal survival and actively orchestrate axon pathfinding, target innervation, and synapse formation through trophic and structural support, ultimately shaping the functional architecture of the PNS [2,3].

Importantly, Schwann cells are not merely passive supporters of neuronal function but dynamic regulators of the peripheral nerve microenvironment, playing pivotal roles in a wide spectrum of pathological conditions. These include injury-induced nerve regeneration, neurodegenerative disorders, neuropathic pain, and even cancer [4,5,6,7]. The dysregulation of Schwann cell functions has been implicated in a range of human hereditary peripheral neuropathies, among which Charcot–Marie–Tooth disease (CMT) is the most prevalent form of hereditary demyelinating neuropathy, with mutations of myelin-related proteins (PMP22, MPZ and Cx32) [8,9,10]. Patients with CMT1 experience progressive distal muscle weakness, sensory loss, and markedly reduced nerve conduction velocity, reflecting underlying morphological abnormalities that include the reduced density of myelinated fibers, pathological demyelination, hypomyelination, tomacula, and “onion bulb” formations, which are hallmark features often leading to severe disability [11,12]. Despite significant advances in genetic diagnosis, there is currently no effective treatment or cure for this condition. Notably, the aberrant activation of intracellular signaling cascades, particularly the MAPK/ERK pathway, has emerged as a convergent mechanism underlying impaired myelination and Schwann cell dedifferentiation in multiple CMT subtypes [13,14]. Despite these advances, how extracellular cues from the nerve microenvironment are sensed and transduced into balanced pro-myelinating signals remains poorly understood.

Discoidin domain receptor 1 (DDR1), a collagen-activated receptor tyrosine kinase (RTK), has recently been identified within the Schwann cell lineage [15,16,17]. Unlike canonical RTKs that respond to soluble growth factors, DDR1 is uniquely activated by fibrillar collagens, the primary structural components of the SC basal lamina [18,19]. *In vitro* studies indicate that phosphorylated DDR1 modulates cell differentiation, migration, and matrix remodeling through downstream signaling cascades, including MAPK/ERK [20,21]. However, the *in vivo* functional role of DDR1 in SC development and PNS myelination has remained unclear.

In this study, we demonstrate that DDR1 is indispensable for Schwann cell differentiation and myelin formation in the mammalian PNS. Using *Ddr1*^−/−^ mice, we reveal that *Ddr1* deficiency results in decreased myelinating Schwann cells, profound hypomyelination, and functional deficits. Mechanistically, *Ddr1* promotes myelination by attenuating the hyperactivation of the MAPK/ERK signaling pathway. These findings implicate DDR1 dysfunction as a potential contributor to the pathogenesis of demyelinating neuropathies, positioning DDR1 as both a fundamental player in PNS development and a promising candidate for future mechanistic and therapeutic investigations in inherited peripheral nerve disorders.

## 2. Results

### 2.1. DDR1 Is Expressed in SCs and Its Loss Leads to Peripheral Nerve Hypoplasia

**Figure 1:**
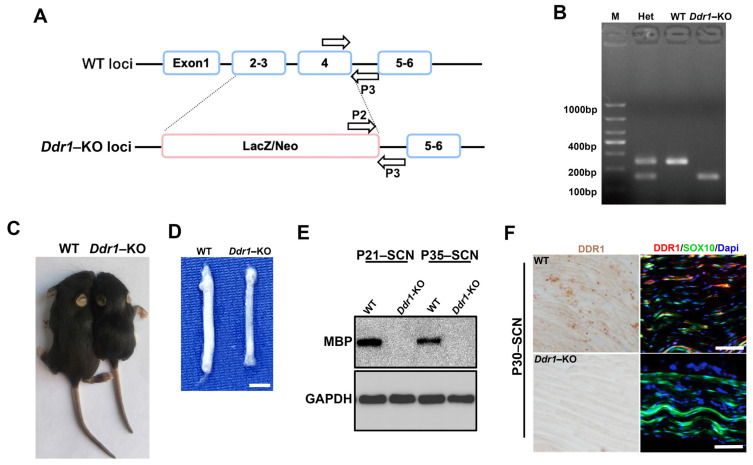
Generation and characterization of *Ddr1*-KO mice. (**A**) Schematic illustration of targeting construct for *Ddr1*-KO mouse generation. Exons 2–4 of the *Ddr1* gene are replaced by LacZ/Neo cassette, resulting in gene disruption. Genotyping was performed using primers P1, P2, and P3. (**B**) Agarose gel electrophoresis of PCR products from WT, heterozygous (Het), and *Ddr1*-KO mice. M: DNA marker; WT: 159 bp; *Ddr1*-KO: 243 bp; Het: both bands present. (**C**) Comparative morphological features of P7 WT and *Ddr1*-KO Mice. *Ddr1*-KO mice exhibit smaller body size, a hunched posture, and thinner fibulae compared to their WT littermates. (**D**) Sciatic nerves from P21 WT and *Ddr1*-KO mice. *Ddr1*-KO mice displayed visibly thinner sciatic nerves compared to WT mice. Scale bar = 10 mm. (**E**) Western blot analysis of sciatic nerve lysates at P21 and P35. DDR1 protein (~120 kDa) was detected in WT sciatic nerve lysates but was completely absent in *Ddr1*-KO samples. GAPDH was used as protein loading control. (**F**) Immunohistochemical staining showed DDR1 expression in sciatic nerve, and immunofluorescence result showed DDR1 co-localizing with SOX10^+^ myelinating Schwann cells. Scale bar = 10 µm.

To visualize DDR1 expression and function in SCs, *Ddr1*-deficient (*Ddr1*^−/−^) mice were utilized, in which the exons 2–4 of the *Ddr1* gene were replaced by a LacZ/Neo cassette (Figure 1A). Genotyping confirmed the expected fragments: 159 bp for wild-type (WT), 243 bp for KO, and both for heterozygous mice (Figure 1B). Phenotypically, *Ddr1*-KO mice exhibited reduced body size, lower body weight, thinner fibulae, and visibly thinner sciatic nerves compared to WT littermates (Figure 1C,D), consistent with previous reports [22]. Western blot confirmed the absence of DDR1 protein (~120 kDa) in KO sciatic nerves at P21 and P35 (Figure 1E). In WT mice, immunohistochemistry (IHC) and immunofluorescence (IF) staining revealed that DDR1 protein was specifically detected in the sciatic nerves, and IF results showed DDR1 with its strong signal co-localizing with the SOX10, confirming its expression in myelinating Schwann cells. Consistently no specific DDR1 protein signal was detected in tissues from *Ddr1*-knockout mice by either IF or IHC (Figure 1F). This structural phenotype suggests that DDR1 may play a potential role in the maturation process of SCs and/or the formation of the myelin sheath during the developmental stages of peripheral nerves.

### 2.2. Ddr1 Mutation Results in a Decrease Expression of Myelin Basic Protein in the Sciatic Nerve

**Figure 2:**
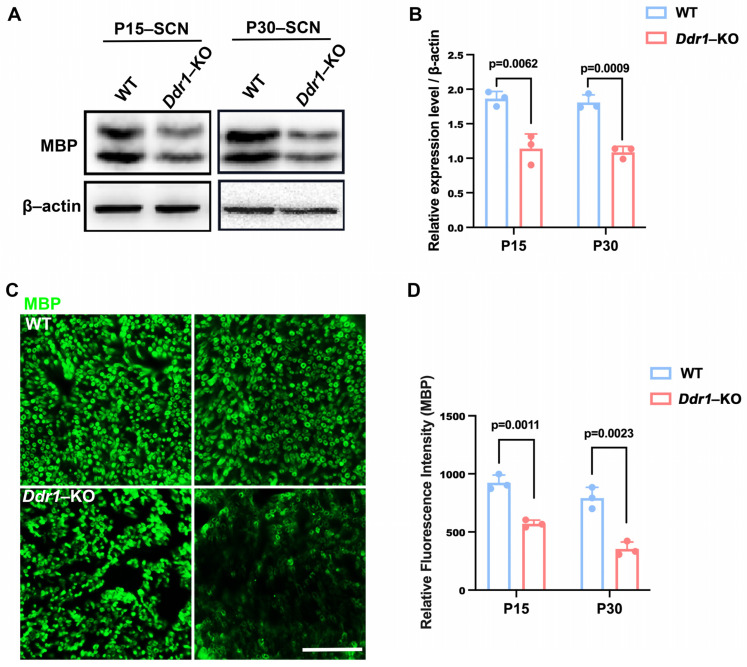
*Ddr1* is expressed in SCs and influences myelin protein levels. (**A**) Western blot showed MBP levels in sciatic nerves of *Ddr1*^−/−^ mice reduced at both P15 and P30 compared to WT littermates. (**B**) Quantification of MBP levels in *Ddr1*-KO and WT sciatic nerves at P15 and P30. Error bar indicates means ± SEM (n = 3). (**C**) Immunofluorescence staining confirms MBP at P15 and P30. Scale bar = 20 µm. (**D**) Quantification of MBP fluorescence intensity in *Ddr1*-KO and WT sciatic nerves. Error bar indicates means ± SEM (n = 3).

To assess the functional consequences of DDR1 deficiency on SCs myelination, the expression of myelin basic protein (MBP), a key marker of myelin, was examined. MBP levels in the sciatic nerves of *Ddr1*^−/−^ mice were found to be significantly reduced at both P15 and P30 compared to WT littermates (Figure 2A,B; *p* = 0.0062 and *p* = 0.0009, respectively). Further IF staining against MBP demonstrated that, at both P15 and P30, myelin sheaths in the sciatic nerves of *Ddr1*^−/−^ mice exhibited a significant reduction in MBP intensity (Figure 2C,D; *p* = 0.0011 and *p* = 0.0023, respectively), accompanied by reduced compaction and disorganized arrangement compared to WT controls, reflecting disrupted myelin bundle architecture. These findings collectively indicate that DDR1 deficiency disrupts SCs myelination during PNS development.

### 2.3. DDR1 Deficiency Affects SC Differentiation and Myelination in Developing Sciatic Nerves

**Figure 3:**
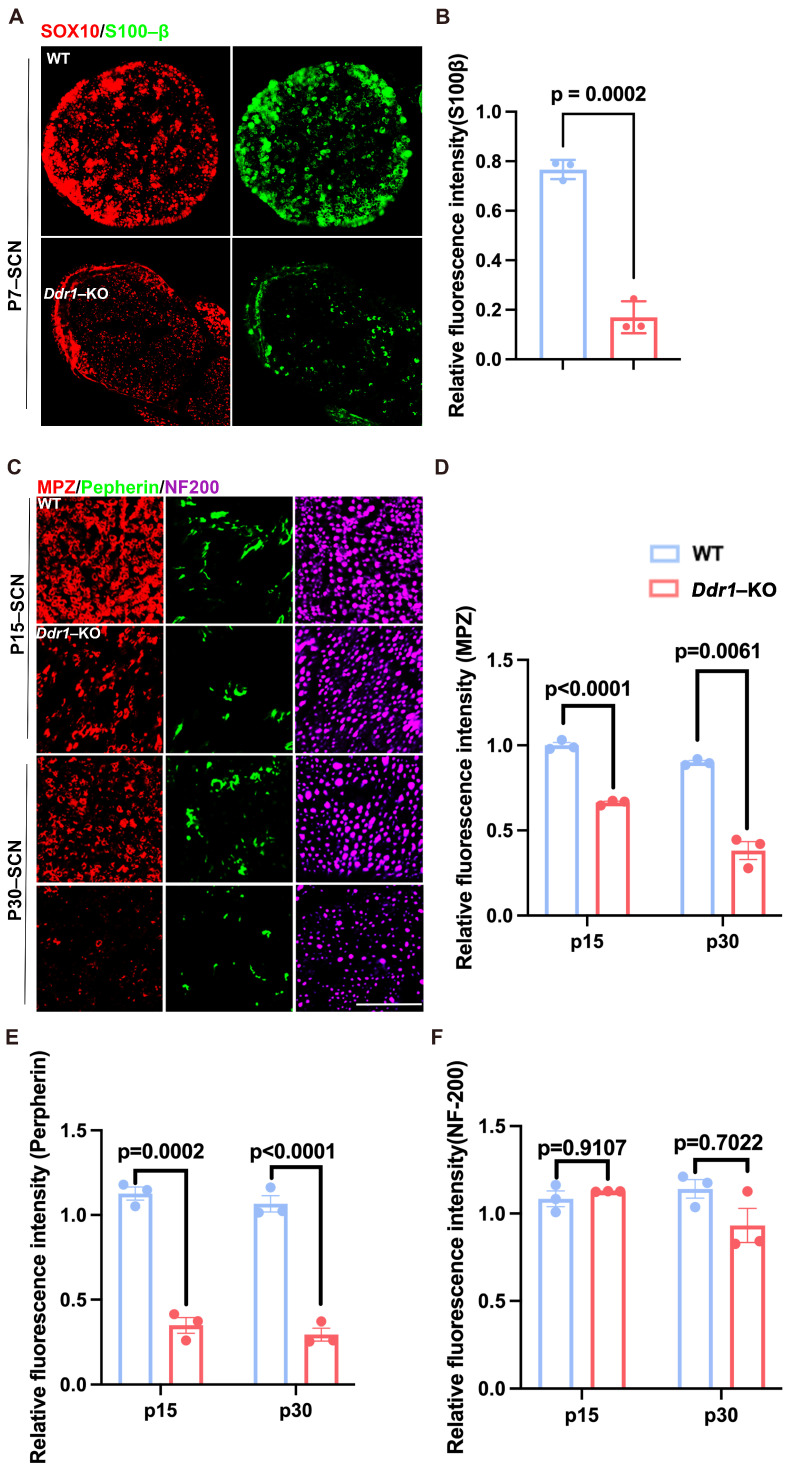
Immunofluorescence analysis reveals that *Ddr1* deficiency impairs differentiation and myelination of Schwann cell in sciatic nerve. (**A**) Double immunostaining for SOX10 (red; immature Schwann cells) and S100β (green; differentiating Schwann cells) in P7 sciatic nerves. Scale bar: 50 µm. (**B**) Quantitative analysis shows significantly reduced S100β^+^ cell numbers in *Ddr1*^−/−^ mice compared to WT littermates (*p* = 0.0002). (**C**) Triple immunofluorescence staining of P15 and P30 sciatic nerve sections: MPZ (red; myelin), peripherin (green; unmyelinated/small-diameter axons), and NF200 (magenta; total axons). Scale bar: 20 µm. (**D**) Quantitative analysis shows significantly reduced MPZ^+^ myelin coverage in *Ddr1*^−/−^ mice compared to WT littermates at P15 (*p* < 0.0001) and P30 (*p* = 0.0061). (**E**) Quantitative analysis reveals decreased peripherin^+^ axon density in *Ddr1*^−/−^ mice at P15 (*p* = 0.0002) and P30 (*p* < 0.0001). (**F**) Quantitative analysis revealed no significant difference in NF200^+^ axon counts between *Ddr1*^−/−^ and wild-type mice at postnatal (P15; *p* = 0.9107) and postnatal day 30 (P30; *p* = 0.7022).

To investigate whether the myelination defect observed in *Ddr1*-deficient mice results from impaired Schwann cell differentiation, we next assess Schwann cell differentiation status. Immunofluorescence staining for SOX10 and S100β was performed at P7. SOX10 is expressed in Schwann cell precursors and immature Schwann cells, whereas S100β is upregulated upon commitment to the myelinating lineage. However, the number of S100β^+^ cells was significantly reduced in *Ddr1*^−/−^ sciatic nerves compared to WT controls (Figure 3A,B, *p* = 0.0002).

To further confirm the role of DDR1 in PNS myelination, immunofluorescence staining for Myelin protein zero (MPZ/P0, SC-specific marker), neurofilament 200 (NF200, axonal marker), and peripherin (unmyelinated/small-diameter axons) were performed in sciatic nerve sections at P15 and P30. In *Ddr1*^−/−^ mice, the proportion of MPZ^+^ myelin was significantly reduced compared to WT littermates (P15: *p* < 0.0001; P30: *p* = 0.0061). Similarly, the number of peripherin^+^ axons was significantly decreased at P15 (*p* = 0.0002) and P30 (*p* < 0.0001). In contrast, the total number of NF200^+^ axons exhibited a downward trend without statistical significance in *Ddr1*^−/−^ mice (Figure 3C–F). Therefore, DDR1 regulated the transition of Schwann cells from precursor to mature myelinating lineage, ensuring their competence in axonal recognition, ensheathment, and myelination; loss of DDR1 caused developmental arrest at early differentiation stages, resulting in cell-autonomous and persistent myelination defects.

### 2.4. The Deficiency of Ddr1 Impairs Axonal Myelination in the Sciatic Nerve

**Figure 4:**
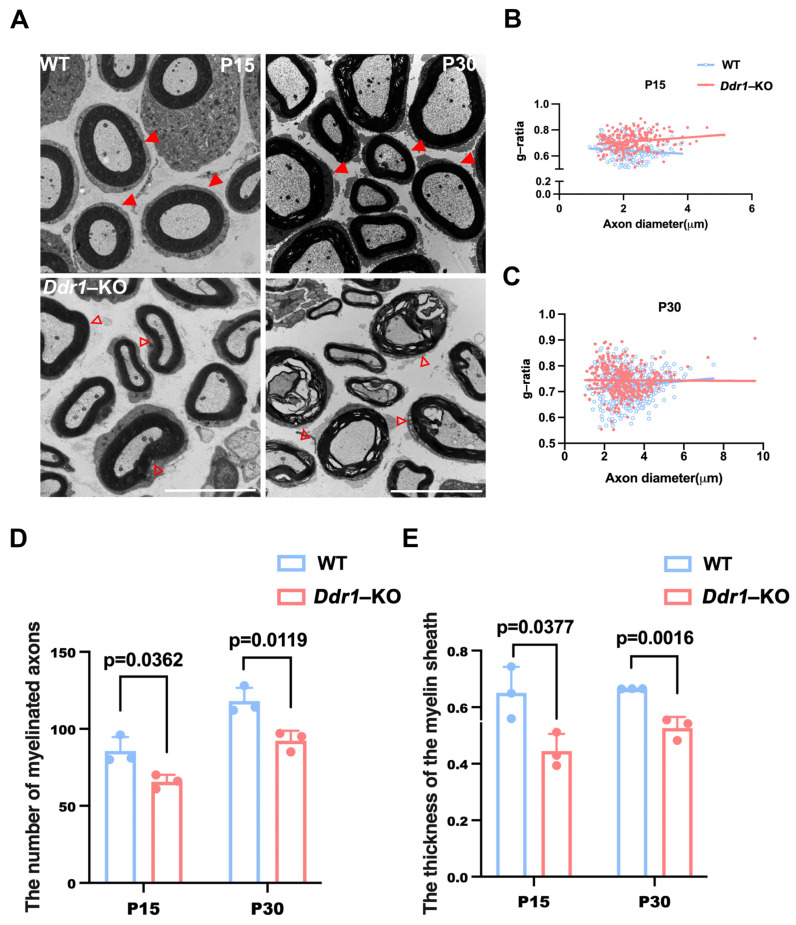
Knockout *Ddr1* alters the myelin structure and myelinated axon constitution in the sciatic nerves. (**A**) Transmission electron microscopy (TEM) images depicting the ultrastructure of sciatic nerves obtained from WT and *Ddr1-KO* mice at P15 and P30. Red solid arrows indicate normal compact myelin sheaths in WT mice, while red hollow arrows point to disrupted and decompacted myelin sheaths in *Ddr1*-KO mice. Scale bar: 5 µm. (**B**,**C**) Scatter plots of the g-ratio (axon diameter/total fiber diameter) versus axon diameter at P15 and P30. At P15, *Ddr1*^−/−^ mice show elevated g-ratio across all axon sizes, indicating thinner myelin. At P30, while most axons maintain a higher g-ratio, a subset of large-diameter axons (>7 μm) exhibit lower g-ratio values. (**D**) Quantitative analysis of the number of myelinated axons. The results demonstrate a significant reduction in *Ddr1*^−/−^ mice compared to WT at both P15 (*p* = 0.0362) and P30 (*p* = 0.0119). Error bars indicate means ± SEM. n ≥ 3. (**E**) Quantitative analysis of myelin sheath thickness. The findings reveal a significant reduction in *Ddr1*^−/−^ mice compared to WT at both P15 (*p* = 0.0377) and P30 (*p* = 0.0016). Error bars indicate means ± SEM. n ≥ 3.

The early effects of *Ddr1* mutation on SC differentiation and myelin gene expression in the sciatic nerve have suggested its important role in myelination. To investigate this possibility, we examined axonal myelination by TEM at different developmental stages. Transmission electron microscopy (TEM) analysis at P15 and P30 revealed irregular, thinly myelinated axons in *Ddr1*-KO mice, with reduced packing density and smaller axonal diameters (Figure 4A). By P30, focal “onion bulb”-like structures, which are indicative of aberrant remyelination, were observed in KO nerves (Figure 4A). Quantitative analysis using Image J revealed that the number of myelinated axons was significantly lower in *Ddr1*^−/−^ mice than in WT controls at both P15 (*p* = 0.0362) and P30 (*p* = 0.0119) (Figure 4D). The g-ratio, defined as the ratio of axonal diameter to total fiber diameter, was significantly higher in *Ddr1*^−/−^ mice compared to WT at P15 (Figure 4B), confirming a relative deficit in myelin thickness for a given axon size. Although *Ddr1*^−/−^ mice exhibited a higher average g-ratio at P30, suggesting generalized myelin deficiency, a subset of large-diameter axons (>7 μm) showed reduced g-ratio values, indicating relatively thicker myelin sheaths compared to WT (Figure 4C), suggesting relative hypermyelination in this population. Moreover, myelin sheath thickness was also markedly reduced in *Ddr1*^−/−^ mice at P15 (*p* = 0.0377) and P30 (*p* = 0.0016) (Figure 4E).

### 2.5. DDR1 Regulates SCs Myelination by Suppressing ERK Signaling in Peripheral Nerves

**Figure 5:**
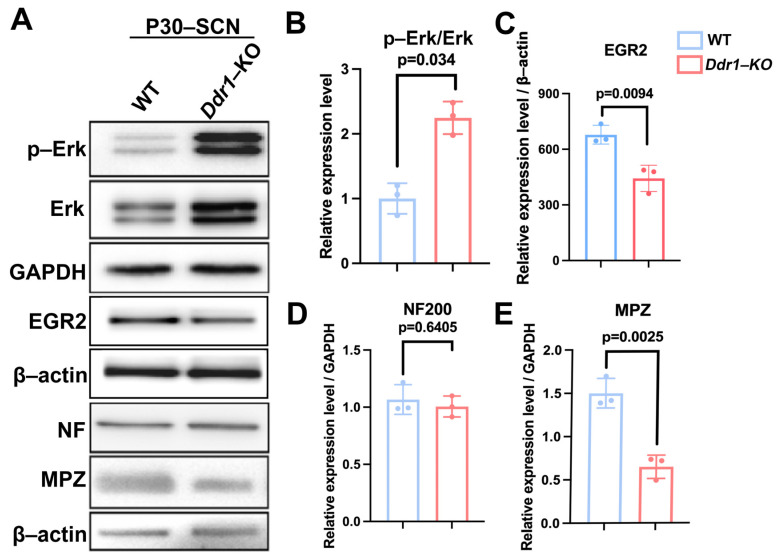
Activation status of signaling pathways and expression of myelination-related proteins in sciatic nerves of WT and *Ddr1*-KO Mice. (**A**) Representative Western blot analysis of sciatic nerve lysates from WT and *Ddr1*^−/−^ mice at P30. Results show increased phosphorylated ERK (p-ERK) expression, decreased MPZ levels, and unchanged NF200 levels in *Ddr1*^−/−^ mice compared to WT controls. (**B**) Quantification of p-ERK/ERK ratio, confirming significantly elevated ERK phosphorylation in *Ddr1*^−/−^ mice (*p* = 0.034), indicating ERK hyperactivation upon DDR1 loss. (**C**) Quantification of EGR2 protein level normalized to β-actin, showing significant downregulation of EGR2 in *Ddr1*^−/−^ sciatic nerves (*p* = 0.0094). (**D**) Quantification of NF200 normalized to GAPDH, showing no significant difference between WT and *Ddr1*^−/−^ mice (*p* = 0.6405), indicating intact axons in absence of DDR1. (**E**) Quantification of MPZ normalized to GAPDH, showing significant reduction in MPZ in *Ddr1*^−/−^ mice (*p* = 0.0025), reflecting impaired myelin synthesis. All data are presented as mean ± SEM; n = 3 per group. Statistical significance was determined by unpaired Student’s *t*-test.

The extracellular signal-regulated kinase (ERK) pathway is a well-recognized regulator of glial differentiation and myelination [23,24,25]. Our previous research has demonstrated that *Ddr1* modulates intracellular signaling via the ERK pathways during central nervous system (CNS) myelination [22]. This notion prompted us to explore the potential role of *Ddr1* in the PNS. To investigate this, we analyzed sciatic nerves from wild-type (WT) and *Ddr1*^−/−^ mice at P30. Western blotting revealed a significant increase in the p-ERK/ERK ratio in *Ddr1*^−/−^ mice (*p* < 0.034; Figure 5A,B), indicating a hyperactivation of ERK phosphorylation following the loss of DDR1. Concomitantly, this dysregulation was accompanied by a marked reduction in myelin protein zero (MPZ) expression (*p* = 0.0025; Figure 5E), a key structural component of peripheral myelin, underscoring the impaired myelin gene expression. Consistent with the impaired myelination phenotype and reduced MPZ expression, we detected a significant downregulation of EGR2 (Krox20), the master transcription factor that directly drives myelin structural gene expression, in the sciatic nerves of *Ddr1*^−/−^ mice compared with WT littermates (Figure 5C). This result indicates that DDR1 deficiency-induced ERK hyperactivation suppresses the expression of core myelination transcription factors, thereby impairing the myelin synthesis program of Schwann cells. In contrast, the expression level of neurofilament 200 (NF200) remained unchanged (*p* = 0.6405; Figure 5D), suggesting that the observed myelination defects are not secondary to axonal degeneration but rather reflect intrinsic Schwann cell dysfunction.

### 2.6. Motor Function Impairment in Ddr1^−/−^ Mice

**Figure 6:**
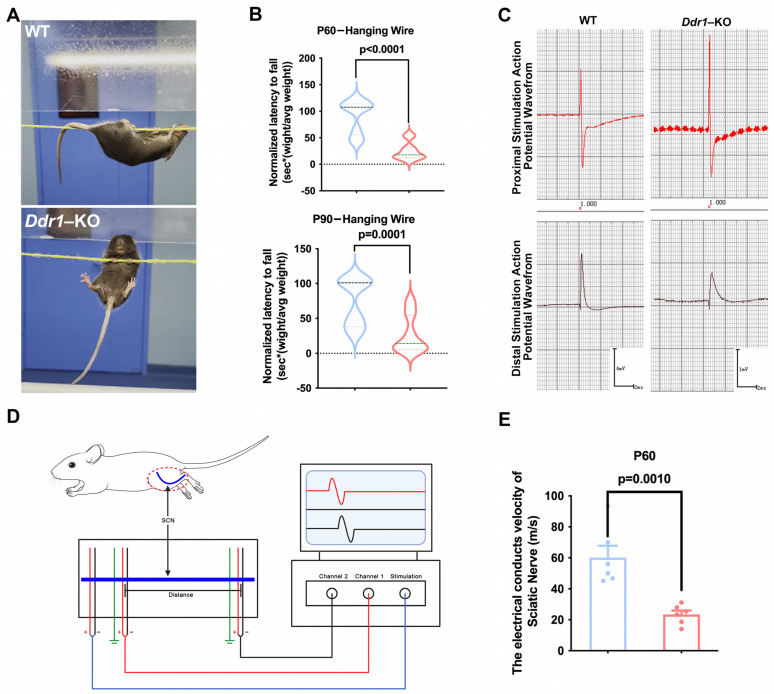
*Ddr1* deficiency impairs motor function and nerve conduction *in vivo*. (**A**) Representative images of WT and *Ddr1*^−/−^ mice during the hanging wire test. (**B**) Normalized latency to fall (sec × (weight/avg weight)) in the hanging wire test at P60 (*p* < 0.0001) and P90 (*p* = 0.0001). (**C**) Representative compound action potential (CAP) waveforms from proximal and distal stimulation of sciatic nerve, showing reduced amplitude and prolonged duration in *Ddr1*^−/−^ mice. (**D**) Schematic illustration of electrophysiological setup for measuring sciatic nerve conduction velocity. The red dashed line indicates the region of the sciatic nerve, and the blue solid line indicates the sciatic nerve itself. (**E**) Electrical conduction velocity of sciatic nerve at P60 is significantly reduced in *Ddr1*^−/−^ mice (*p* = 0.0010). Data are mean ± SEM; n ≥ 5 per group; unpaired Student’s *t*-test.

Myelination within the PNS plays a pivotal role in the precise regulation of motor functions. To ascertain whether the absence of *Ddr1* contributes to motor function impairment, we evaluated motor performance at P60 and P90 using the hanging wire test (Figure 6A). *Ddr1*^−/−^ mice exhibited significantly reduced normalized latency to fall compared to their WT littermates at P60 (*p* < 0.0001) and P90 (*p* = 0.0001; Figure 6B). These results indicate impaired neuromuscular coordination and grip strength, consistent with compromised peripheral nerve function. This motor dysfunction was observed to be persistent across the evaluated time points.

To further investigate the electrophysiological basis of this dysfunction, we performed nerve conduction studies on the sciatic nerve. Representative compound action potentials (CAPs) recorded upon proximal and distal stimulation revealed abnormal waveform morphology in *Ddr1*^−/−^ mice, including reduced amplitude and prolonged duration (Figure 6C). These changes are indicative of impaired axonal excitability and disrupted signal propagation. Quantitative analysis of the electrical conduction velocity (ECV) of the sciatic nerve showed a significant reduction in *Ddr1*^−/−^ mice at P60 (*p* = 0.00010; Figure 6E), consistent with defective myelination and slowed nerve impulse transmission. The schematic in Figure 6D illustrates the experimental setup for measuring ECV, where stimuli are applied at two sites along the sciatic nerve, and the time difference between evoked responses is used to calculate conduction velocity. Together, these findings demonstrate that a loss of DDR1 leads to measurable motor deficits and impaired nerve conduction, directly linking the molecular and structural abnormalities in Schwann cells to functional impairments in the peripheral nervous system physiology.

## 3. Discussion

Discoidin domain receptor 1 (DDR1) is a collagen-activated receptor tyrosine kinase implicated in diverse physiological and pathological processes, including fibrosis, inflammation, cancer, and nervous system disorders [26,27,28]. While DDR1 expression has been well documented in epithelial cells and multiple tissues such as the brain, lung, kidney, and skin [21,22,29,30], a tissue-based map of the human proteome systematically profiled the expression distribution of human proteins including DDR1 across 32 human tissues, confirming that the tissue expression profile of functional proteins like DDR1 provides a panoramic reference framework for understanding its multi-tissue physiological functions. This study also systematically analyzed the tissue distribution characteristics of drug targets such as DDR1, providing fundamental data for subsequent tissue-specific intervention research [31]. Its functional role in the PNS (particularly in Schwann cell biology) has not been explored. A recent cross-species integration study constructed harmonized cell atlases of trigeminal and dorsal root ganglia, systematically resolving the composition and cell-cell interaction characteristics of 18 neuronal subtypes and 11 non-neuronal cell types (including Schwann cells, immune cells, etc.) in the peripheral sensory nerve region, providing a unified reference system for the cell-level research of the peripheral nerve microenvironment [32]. SCs originate from neural crest precursors and undergo a tightly orchestrated developmental program, culminating in the bifurcation of myelinating or non-myelinating SCs [3,4,33]. Notably, peripherin, a class III intermediate filament specifically expressed in small-diameter axons ensheathed by non-myelinating SCs (Remak bundles), serves as a sensitive marker for this axonal population. In our *Ddr1*^−/−^ model, peripherin-positive axons were significantly reduced at both P15 and P30, while NF200, a marker of large-diameter myelinated axons, showed only a non-significant downward trend. This selective vulnerability suggests that Remak bundle formation and maintenance are particularly sensitive to Schwann cell dysfunction, reinforcing the critical role of DDR1 in non-myelinating SC differentiation and axon–glia interaction. Their fate decision is largely dictated by axonal cues, most notably Neuregulin-1 (NRG1) signaling from large-diameter axons (>1 µm) [34,35]. Beyond myelin production, SCs provide trophic support, maintain the basal lamina, and orchestrate nerve regeneration functions that depend critically on their proper differentiation and maturation [3,4].

Recent studies have detected DDR1 expression in central glia, where it modulates oligodendrocyte differentiation and CNS myelination [22,30,36]. In the PNS, DDR1 has been identified as a transcriptional target of SOX10 and is dysregulated in schwannomas [16,37], hinting at a potential role in SC development. However, the *in vivo* function of DDR1 in peripheral myelination had not been established.

**Figure 7:**
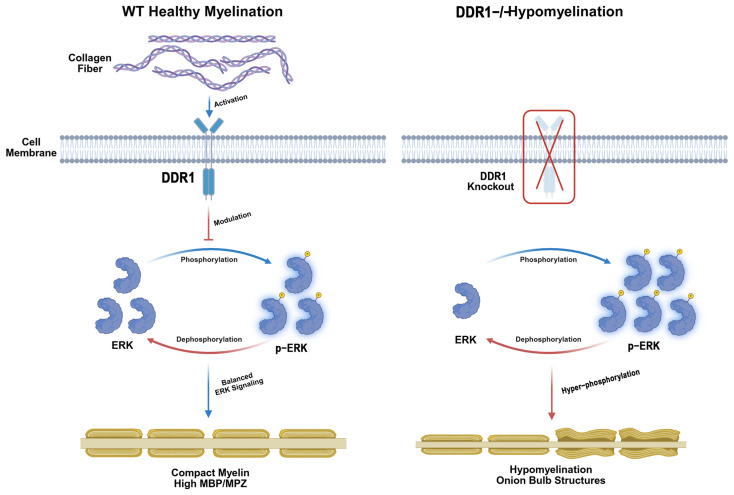
Schematic model of the DDR1-ERK signaling axis in Schwann cell myelination. Under physiological conditions (**Left**), DDR1 functions as a vital mechanosensory link that senses the extracellular collagen matrix and acts as a “molecular brake” to constrain ERK phosphorylation. This homeostatic control ensures the timely initiation of the myelination program and the assembly of compact myelin sheaths. In the pathological state (DDR1 knockout, (**Right**)), the absence of this regulatory brake triggers persistent ERK hyperactivation, which prevents Schwann cells (SCs) from transitioning to a mature phenotype. This differentiation arrest leads to the downregulation of myelin genes (e.g., *Mpz*, *Mbp*), resulting in the characteristic hypomyelination and focal “onion bulb” formations observed in *Ddr1*^−/−^ nerves.

Here, we provide genetic evidence that DDR1 is essential for Schwann cell differentiation and peripheral nerve myelination. *Ddr1*^−/−^ mice exhibit profound hypomyelination, characterized by the reduced expression of key myelin proteins MPZ, MBP, and peripherin, as well as abnormal ultrastructural features such as thinly wrapped sheaths, irregular compaction, and focal “onion bulb” formations reminiscent of human demyelinating neuropathies like CMT1 [11,38,39]. These structural deficits are accompanied by functional impairments: *Ddr1*^−/−^ mice display significant motor dysfunction in the hanging wire test and markedly slowed sciatic nerve conduction velocity, directly linking DDR1 loss to compromised PNS physiology. To integrate these phenotypic, functional, and mechanistic findings into a unified working model, the DDR1-ERK signaling axis is delineated, with its central role in governing Schwann cell maturation highlighted (Figure 7). In this model, DDR1 is proposed to act as a critical mechanosensor that restrains aberrant ERK hyperactivation, functioning as a “molecular brake” to ensure the ordered progression of the myelination program. Ablation of this regulatory brake triggers a cascade of events that arrest Schwann cell differentiation, ultimately driving the structural and functional deficits observed in *Ddr1*^−/−^ peripheral nerves.

Mechanistically, we demonstrate that DDR1 deficiency leads to the hyperactivation of the MAPK/ERK signaling pathway in developing sciatic nerves, as evidenced by elevated p-ERK/ERK ratios at both P15 and P30. This ERK overactivation coincides with the downregulation of myelin genes and peripherin expression. The reduction in peripherin, a marker for unmyelinated/small-diameter axons [40], indicates that non-myelinating SCs fail to properly ensheathe small-diameter axons, and that SCs exhibit a compromised recognition capacity for axons of all calibers. Notably, axonal integrity, as assessed by neurofilament 200 (NF200) expression [41], remains intact, confirming that the observed myelination defects are intrinsic to SCs rather than secondary to axonal degeneration. We further show that DDR1 acts as a key mechanotransducer, converting structural and spatial cues from the extracellular collagen matrix into intracellular regulatory signals. This collagen-DDR1 axis enables SCs to sense the maturation state of the surrounding basal lamina, thereby precisely calibrating the timing of myelin sheath expansion to match the developmental progression of the peripheral nerve [15,42]. Our findings align with and extend our prior work in the CNS, where DDR1 was shown to promote oligodendrocyte differentiation via the suppression of ERK signaling [22]. The current data reveal a conserved DDR1-ERK regulatory axis across both central and peripheral glia. Functional studies on human genetic disorders revealed that the loss-of-function of DDR1 causes chondrodysplasia with multiple dislocations. Mechanistically, DDR1 regulates the differentiation and matrix synthesis function of chondrocytes and osteoblasts by modulating p38 MAPK, IHH, and non-canonical WNT signaling pathways, indicating that the regulatory effect of DDR1 on MAPK family signaling pathways is conserved across multi-tissue development [43]. In this context, DDR1 serves as a specialized “molecular brake” that constrains ERK signaling intensity within a precise physiological window. By preventing signal “overflow,” DDR1 ensures that Schwann cells transition smoothly from a promyelinating state to active myelin synthesis. Mechanistically, the hyperactivation of ERK signaling caused by DDR1 deletion may downregulate the core myelination master transcription factor EGR2, which in turn inhibits the expression of myelin structural genes and the normal myelination program of Schwann cells. Given that aberrant ERK activation is a convergent mechanism in multiple forms of CMT [13,14], DDR1 dysfunction may represent a novel pathogenic contributor to inherited demyelinating neuropathies.

The identification of this DDR1-ERK regulatory axis also highlights potential therapeutic targets for the treatment of demyelinating peripheral neuropathies. Currently, the clinical management of inherited neuropathies, such as CMT disease, remains largely supportive, centered on rehabilitation and symptomatic pain management, as there are still no approved disease-modifying therapies [44,45]. Our findings suggest that targeting key regulators of Schwann cell differentiation, specifically, the DDR1-mediated negative regulation of ERK signaling represents a promising therapeutic strategy to develop disease modifying interventions, moving beyond purely symptomatic treatment.

Given that DDR1 ablation drives pathological ERK hyperactivation, strategies to restore DDR1 function, or rebalance its downstream signaling, hold substantial translational potential [22,28,46,47]. The successful use of AAV-delivered CRISPR/Cas9 systems for *in vivo* point mutation correction exemplified by studies in familial hypercholesterolemia models, where the partial rescue of LDLR expression effectively ameliorated disease phenotypes, provides a well-established technical framework for this approach [48]. Accordingly, similar gene correction strategies could be adapted to target pathogenic DDR1 variants identified in human clinical sequencing databases, with the potential to restore functional receptor expression and activity in patient SCs. In summary, this study identifies DDR1 as a novel regulator of peripheral myelination that acts by restraining ERK signaling. These findings advance our understanding of Schwann cell biology and open new avenues for treating demyelinating diseases.

## 4. Materials and Methods

### 4.1. Animals

*Ddr1*-knockout (*Ddr1*-KO) mice (LEXKO-1928) were obtained from Lexicon Pharmaceuticals Incorporated (The Woodlands, TX, USA) and their genetic background has been characterized in earlier studies [22]. The animals were maintained under controlled environmental conditions with a constant temperature of 22 ± 1 °C and a 12 h light/dark cycle. In this study, all animal protocols were carried out in compliance with institutional ethical standards and were formally approved by the Laboratory Animal Center and the Animal Ethics Committee of Hangzhou Normal University, China (Approval No. 2022-1063; approved 3 March 2022).

Genomic DNA was extracted from mouse tail tips and used for genotyping via polymerase chain reaction (PCR). The primers used for identifying the genotypes of *Ddr1*-knockout (*Ddr1*-KO) mice were as follows: P1: 5′-GTTGCGTTACTCCCGAGATG-3′; P2: 5′-GCAGCGCATCGCCTTCTATC-3′; and P3: 5′-AGACAATCTCGAGATGCTGG-3′. Following intraperitoneal anesthesia with Avertin (Sigma-Aldrich, St. Louis, MO, USA, T48402), mice were transcardially perfused with 4% paraformaldehyde for fixation. Sciatic nerve tissues were then harvested and subjected to gradient dehydration using 10%, 20%, and 30% sucrose solutions. Finally, the tissues were embedded in optimal cutting temperature (OCT) compound. All experimental groups used mice with a matched male–female sex ratio. Our previous study has verified that there is no sex-dependent difference in the neural developmental phenotypes mediated by *Ddr1* knockout, and no sex-related phenotypic difference was observed in this study.

### 4.2. Immunohistochemistry and Immunofluorescence Staining

Sciatic nerve tissues were collected at defined developmental stages. Mice were deeply anesthetized and transcardially perfused with ice-cold 4% paraformaldehyde (PFA; Sangon, Shanghai, China). Following perfusion, sciatic nerves were carefully dissected and post-fixed overnight at 4 °C. Tissues were then cryoprotected in 30% sucrose solution, embedded in optimal cutting temperature compound (OCT, Cat. No. 6502; Thermo Scientific, Waltham, MA, USA), and sectioned at a thickness of 16 μm using a cryostat.

For immunofluorescence staining, tissue sections were blocked with 10% goat serum in PBS containing 0.2% Triton X-100 (Aladdin, Shanghai, China, T109026) for 1 h at room temperature. Sections were subsequently incubated with primary antibodies overnight at 4 °C. After thorough washing, Alexa Fluor 488- or 594-conjugated secondary antibodies (Invitrogen, Carlsbad, CA, USA) were applied for 2 h at room temperature. Nuclei were counterstained with DAPI, and sections were mounted using Mowiol mounting medium (containing Mowiol 4-88, Sigma-Aldrich, St. Louis, MO, USA). Fluorescent images were acquired using a ZEISS Imager.M2 microscope (Zeiss, Oberkochen, Germany). Detailed information regarding the primary antibodies used is provided in Table 1.

For immunohistochemistry staining, sections were first incubated in a solution of 80% methanol containing 0.6% hydrogen peroxide (H_2_O_2_) for 30 min to quench endogenous peroxidase activity. Sections were then rinsed in 1× PBS and blocked with 5% goat serum for 1 h at room temperature. The anti-DDR1 antibody (1:800, #5583T, Cell Signaling Technology, Danvers, MA, USA) was applied overnight at 4 °C. After washing, a biotinylated secondary antibody (1:1000, Vector Laboratories, Newark, CA, USA) was added, followed by incubation with ABC reagent (Vector Laboratories, Newark, CA, USA) and development with DAB chromogen solution. Stained sections were imaged using a ZEISS Imager.M2 microscope (Zeiss, Oberkochen, Germany).

### 4.3. Transmission Electron Microscopy (TEM)

The sections were collected from the sciatic nerve from P15 to P60. Mice were perfused with a phosphate buffer solution containing 2.5% glutaraldehyde and 4% PFA. The sciatic nerve tissues were isolated and post-fixed in 1% osmium tetroxide for 1 h. The tissues were then washed in a 0.1 M cacodylate buffer, dehydrated in graded ethanol, and embedded in epoxy resins. Ultrathin sections (0.5 µm) were stained with toluidine blue and observed under a transmission electronic microscope. All quantitative analyses were performed in a strictly blinded manner to avoid subjective bias. For each mouse, we quantified 400 axons with intact structure and clear boundaries, with at least three biological replicates per genotype. The g-ratio was calculated as the ratio of inner axonal diameter to total outer diameter of the myelinated fiber, while myelin sheath thickness was directly measured based on the scale bar of TEM images by using Image J (version 1.53).

### 4.4. Western Blot Analysis

Sciatic nerve tissues from mice at different developmental stages were lysed in RIPA Lysis Buffer supplemented with a protease inhibitor cocktail. Lysates were centrifuged at 12,000× *g* and 4 °C for 15 min to get rid of the unsolved debris. The concentration of the supernatant was measured by a BCA assay (23225, Thermo Scientific, Waltham, MA, USA). Proteins in samples were separated by 6–12% SDS-PAGE, transferred to an Immobilon-P Transfer Membrane (Millipore, Kenelworth, NJ, USA) and then incubated with indicated primary antibodies diluted in a blocking buffer at 4 °C overnight after blocking by a 5% non-fat milk solution in TBST (50 mM Tris, pH 7.4, 150 mM NaCl, and 0.1% Tween 20) for 1 h at RT. Protein detection was achieved with an enhanced chemiluminescence system (Amersham Biosciences, Amersham, UK). Detailed information on the primary and secondary antibodies used is provided in Table 1.

### 4.5. Hanging Wire Test

A horizontal rope (diameter: 1 cm; length: 2 m) was suspended parallel to the ground. Each mouse was placed in the center of the rope, and the time until the animal fell or reached either end of the rope was recorded, with a maximum trial duration of 120 s. Three trials were conducted per mouse, with 20 min intervals between each. The average latency to fall was normalized to the mean body weight of mice of the same sex for the final statistical analysis.

### 4.6. Determination of Motor Nerve Conduction Velocity

At postnatal day 60 (P60), sciatic nerves were rapidly dissected from euthanized mice and immediately transferred into ice-cold 1× PBS. The isolated sciatic nerve was carefully mounted onto the recording chamber of a nerve conduction setup. Electrical stimulation was applied, and compound motor action potentials (CMAPs) were recorded via two channels. Biphasic waveforms were observed on both Channel 1 and Channel 2, with a measurable time delay between the two signals. The distance between the two recording electrodes was determined, and the motor nerve conduction velocity (MNCV) was calculated.

### 4.7. Statistical Analysis

All data were analyzed using GraphPad Prism 8 (GraphPad Software, San Diego, CA, USA) and presented as mean ± SEM. The two-way analysis of variance (ANOVA) followed by a multiple comparison was used for the quantitative analysis of data over time and between genotypes. The unpaired two-tailed Student’s *t* test was used for analysis between two groups with one variable. In addition, a *p*-value of <0.05 was considered statistically significant. For each analysis, the results from independent animals were treated as biological replicates (n ≥ 3). For Western blotting and staining results, statistical analyses were performed after the subtraction of the background intensity and normalization with controls in each batch of experiments to minimize the influences of batch-to-batch variations. Detailed statistical information for each experiment was included in the figure legends.

## Figures and Tables

**Table 1:** Antibody Information Table.

Antigen	Species	IF/IHC	WB	Source	Catalog Number
MBP	Mouse	1:500	1:1000	Millipore	MAB382
SOX10	Rabbit	1:500	1:2000	oasisbiofarm	PRB053
DDR1	Rabbit	1:800		cell signaling	5583
β-actin	Mouse		1:10,000	HUABIO	EM21002
S100-β	Mouse	1:500		oasisbiofarm	MMS037-01
MPZ	Rabbit	1:200	1:2000	ABclonal	A21931
PERIPHERIN	Rabbit	1:1000		Abcam	ab246502
NF200	Rabbit	1:3000	1:300	oasisbiofarm	PGP081
ERK	Rabbit	1:500	1:2000	Bioss	bs-3016R
p-ERK	Rabbit		1:5000	Abcam	ab76299
GAPDH	Mouse		1:10,000	ABclonal	AC002
Goat-anti-Rabbit IgG (H+L), 488		1:3000		Invitrogen	A11034
Goat-anti-Rabbit IgG (H+L), 594		1:3000		Invitrogen	A11012
Goat-anti-Rabbit IgG (H+L), HRP			1:5000	Invitrogen	31460
Goat-anti-Mouse IgG (H+L), HRP			1:5000	Invitrogen	31430
Cy3 Goat anti-Mouse IgG		1:500		HUABIO	HA1109

## Data Availability

The original contributions presented in this study are included in the article. Further inquiries can be directed to the corresponding author.
